# T lymphocyte characteristics and immune repertoires in the epicardial adipose tissue of heart failure patients

**DOI:** 10.3389/fimmu.2023.1126997

**Published:** 2023-03-07

**Authors:** Xu-Zhe Zhang, Xian-Li Chen, Ting-Ting Tang, Si Zhang, Qin-Lin Li, Ni Xia, Shao-Fang Nie, Min Zhang, Zheng-Feng Zhu, Zi-Hua Zhou, Nian-Guo Dong, Xiang Cheng

**Affiliations:** ^1^ Department of Cardiology, Union Hospital, Tongji Medical College, Huazhong University of Science and Technology, Wuhan, China; ^2^ Hubei Key Laboratory of Biological Targeted Therapy, Union Hospital, Tongji Medical College, Huazhong University of Science and Technology, Wuhan, China; ^3^ Hubei Provincial Engineering Research Center of Immunological Diagnosis and Therapy for Cardiovascular Diseases, Union Hospital, Tongji Medical College, Huazhong University of Science and Technology, Wuhan, China; ^4^ Department of Cardiovascular Surgery, Union Hospital, Tongji Medical College, Huazhong University of Science and Technology, Wuhan, China

**Keywords:** epicardial adipose tissue, heart failure, immune infiltration, T lymphocytes, TCR immune repertoires, bioinformatics analyses

## Abstract

**Background:**

Epicardial adipose tissue (EAT) acts as an active immune organ and plays a critical role in the pathogenesis of heart failure (HF). However, the characteristics of immune cells in EAT of HF patients have rarely been elucidated.

**Methods:**

To identify key immune cells in EAT, an integrated bioinformatics analysis was performed on public datasets. EAT samples with paired subcutaneous adipose tissue (SAT), heart, and peripheral blood samples from HF patients were collected in validation experiments. T cell receptor (TCR) repertoire was assessed by high-throughput sequencing. The phenotypic characteristics and key effector molecules of T lymphocytes in EAT were assessed by flow cytometry and histological staining.

**Results:**

Compared with SAT, EAT was enriched for immune activation-related genes and T lymphocytes. Compared with EAT from the controls, activation of T lymphocytes was more pronounced in EAT from HF patients. T lymphocytes in EAT of HF patients were enriched by highly expanded clonotypes and had greater TCR clonotype sharing with cardiac tissue relative to SAT. Experiments confirmed the abundance of IFN-γ^+^ effector memory T lymphocytes (T_EM_) in EAT of HF patients. CCL5 and GZMK were confirmed to be associated with T lymphocytes in EAT of HF patients.

**Conclusion:**

EAT of HF patients was characterized by pronounced immune activation of clonally expanded IFN-γ^+^ T_EM_ and a generally higher degree of TCR clonotypes sharing with paired cardiac tissue.

## Introduction

Due to its unique anatomic and functional features (1), epicardial adipose tissue (EAT) and its critical role in the pathogenesis of cardiovascular diseases have received increasing attention in recent years. EAT covers nearly 80% of the heart’s surface and accounts for approximately 15% of the total heart mass (2). EAT is mainly located in the atrioventricular and the interventricular sulcus (3). EAT is in direct contact with the myocardium without fascial interruption, allowing mutual crosstalk. Under normal conditions, EAT is cardio-protective by maintaining lipid homeostasis and providing mechanical protection to the adjacent myocardium. Under pathological conditions, however, EAT transforms into a pro-inflammatory and pro-fibrotic phenotype and is cardiac deleterious (4).

**Figure 1:**
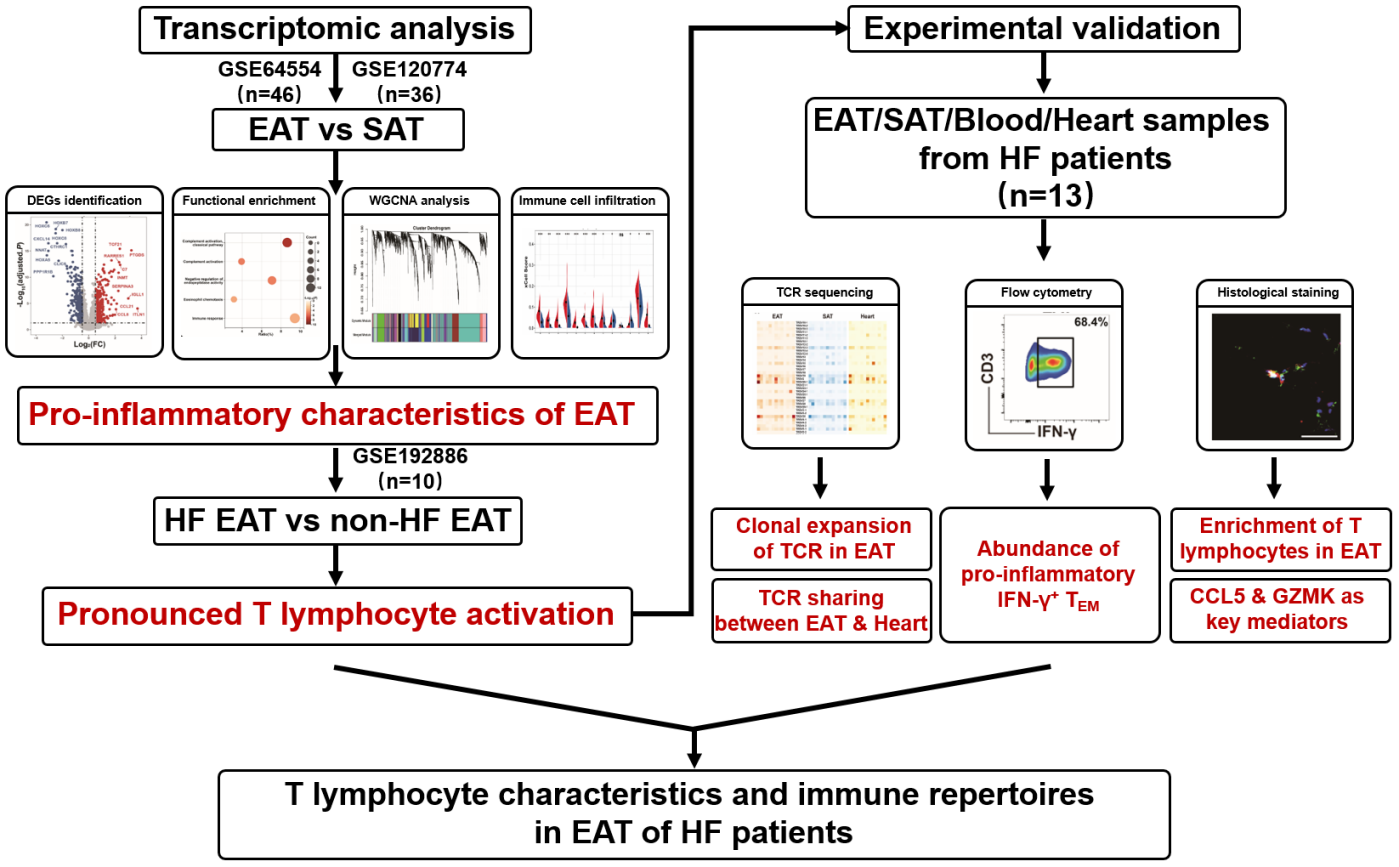
Overall flowchart of this study.

Heart failure (HF) is a complex clinical condition with a poor prognosis characterized by cardiac diastolic or systolic dysfunction (5). Emerging evidence has linked EAT to the pathogenesis of HF (4, 6). Sodium-glucose cotransporter 2 (SGLT2) inhibitors is a novel agent for the treatment of HF (7). A reduction in EAT volume has been linked to the beneficial effects of SGLT2 inhibitor in HF patients (8). The mechanisms by which EAT contributes to HF remain unclear, but likely involve enhanced inflammation. EAT is populated by immune cells including macrophages, T lymphocytes, mast cells, etc., and serves as the source of pro-inflammatory mediators (9–11). Pro-inflammatory cytokines and pro-fibrotic factors, such as leptin, TNF-α, IL-1β, and IL-6 are up-regulated in EAT under pathological conditions (12, 13) and may diffuse into the adjacent myocardium to promote cardiac dysfunction. However, a better understanding of the relationship between EAT and HF requires a full-scale knowledge of the changes in the immune microenvironment within EAT in HF. Here, we performed integrated bioinformatics and immune cell infiltration analyses on public datasets to characterize the immune features and immune cell profiles of EAT in HF patients. The results suggested that EAT of HF patients was characterized by pronounced immune activation, particularly by the accumulation of T lymphocytes. Further analyses indicated that T lymphocytes in EAT of HF patients were highly expanded, closely related to those in cardiac tissue, and dominated by IFN-γ^+^ effector memory T lymphocytes (T_EM_). GZMK and CCL5 identified by bioinformatics analyses may act as the key effector molecules of T lymphocytes in EAT of HF patients. The overall flowchart of this study is shown in **Figure 1**.

## Materials and methods

### Public datasets in transcriptomic analysis

GSE64554 (14), GSE120774 (15), GSE192886 (16) and GSE24425 (17) were obtained from Gene Expression Omnibus (GEO, https://www.ncbi.nlm.nih.gov/geo). Array or sequencing data of paired EAT and SAT in GSE64554 (n=46), GSE120774 (n=36), and GSE24425 (n=12) were from patients undergoing cardiac valve or coronary artery bypass graft surgery. GSE192886 contained sequencing data of EAT from HF patients (n=5) and non-HF patients (n=5) undergoing coronary artery bypass graft surgery. Clinical characteristics for analyzed patients can refer to the original citations of these datasets and **Tables S1**-**S3**.

### Patients and samples in the experimental validation

In the validation experiments, fresh EAT with paired SAT, heart, and peripheral blood samples were collected from HF patients undergoing heart transplantation in Wuhan Union Hospital. Peripheral blood samples were obtained before surgery. SAT samples were obtained from the suprasternal region, heart and EAT samples were obtained from the left ventricle. We obtained informed consent from all enrolled subjects. The experimental protocol and sample collection were in accordance with the Declaration of Helsinki and approved by the Medical Ethics Committee of Wuhan Union Hospital of Huazhong University of Science and Technology (METC number: 20200462). Information on the involved subjects was listed in **Table S4**.

### Identification of differentially expressed genes, functional enrichment analysis, PPI network construction, and identification of hub genes

The data obtained from GSE64554 and GSE120774 were processed by log_2_ transformation and quantile normalization *via* limma package (18) using R separately. The differential expression matrixes of the datasets were also identified by the limma package separately and *P* values were adjusted by the Benjamini-Hochberg method. We then applied the Robust Rank Aggregation (RRA) method (19) to filter the differential expression matrixes, so as to obtain the comprehensive differentially expressed genes (DEGs) across two different microarray platforms. DEGs with RRA score less than 0.05 were selected for further analyses.

Functional enrichment analyses were performed using the DAVID (20) by inputting the official gene symbols of obtained DEGs. Figures for functional enrichment analyses were plotted by R and Sangerbox (http://www.sangerbox.com/tool). Construction of the protein-protein interaction (PPI) network and identification of hub genes were performed as the previous description (21).

### Weighted gene co-expression network analysis

To explore the gene modules responsible for the phenotypic differences between EAT and SAT, we performed the Weighted gene co-expression network analysis (WGCNA)to identify co-expressed gene modules (22). First, we screened the top 25% of the genes in the variance variability between samples in a pooled matrix and used them as input data. Next, we obtained the soft threshold and set the minimum gene number in the module to 30 to get gene co-expression modules. By analyzing the correlation between each module with the EAT/SAT phenotypes, we screened out the gene modules that need further exploration. Finally, functional enrichment analyses were performed on the obtained modules, and the modules significantly related to the immune process were identified. By taking the intersection of immune-related key modules and DEGs identified by RRA, we obtained a set of key immune-related genes.

### Immune cell infiltration and correlation analyses

xCell (23) and CIBERSORT (24) are signature-based methods to infer the immune cell landscape according to expressional profiling. We performed immune cell infiltration analyses and obtained the immune cell landscapes for EAT and SAT based on the pooled matrix. “Lymphoid cells” and “myeloid cells and others” were categorized. Results were evaluated by t-test to determine the significance of differences. The correlation relationship between immune cell types, WGCNA modules, and target genes was evaluated by Pearson correlation coefficients.

### T cell receptor repertoires sequencing and analyses

Paired EAT, SAT, and heart samples were used for T cell receptor (TCR) repertoire sequencing. Tissue genomic DNA was extracted using Universal Genomic DNA Kit (CWBio, China). DNA quality was evaluated using Nanodrop2000 (Thermo, USA) with concentration >20ng/uL and OD260/280 between 1.7 and 2.0. Multiplex PCR reactions were run to specifically amplify the third complementarity-determining region (CDR3) of the TCRβ chain for libraries construction. The constructed libraries were deeply sequenced by Illumina NextSeq500. Primers and sequencing were provided by SEQHealth (China).

Raw sequences filtered by SOAPnuke (version 1.6.0) were used for TCR sequencing analyses, and the sequencing data were mapped to the ImMunoGeneTics (IMGT) database using MiXCR (version 3.0.3) to define the V, D, and J fragments and CDR3 sequence (25). The terms TCR clonotype and TCR clone describe the CDR3 sequence composed of a unique amino acid sequence and CDR3 sequence composed of unique V, D, and J fragments, respectively. Antigen matching analysis was performed *via* the IEDB database (http://www.iedb.org/).

### Flow cytometry

Peripheral blood mononuclear cells (PBMCs) were isolated by density gradient centrifugation using lymphocyte separation medium (MPbio, USA). Fresh EAT samples were digested at 37°C in Hepes buffer containing collagenase D (1mg/mL, Sigma, USA) and dispase II (2mg/mL, Sigma, USA), and then filtered by 100μm and 40μm filters (Falcon, USA) sequentially to collect the stromal vascular fraction (SVF) for subsequent flow cytometric analyses. Memory phenotypes of T lymphocytes were categorized into naïve T cell (T_N_, CD62L^+^CD45RA^+^), central memory T cell (T_CM_, CD62L^+^CD45RA^-^), effector memory T cell (T_EM_, CD62L^-^CD45RA^-^) and CD45RA^+^ effector memory T cell (T_EMRA_, CD62L^-^CD45RA^+^). For the detection of interferon (IFN)-γ, cells were re-suspended in RPMI-1640 medium (Gibco, USA) with 10% heat-inactivated FBS (Gibco, USA) at a concentration of 10^6^ cells/ml and stimulated with Cell Stimulation Cocktail (eBioscience, USA). After 6 hours of stimulation, cells were harvested, permeabilized, and then stained with fluorescence-conjugated antibodies. Used antibodies were as follows: PE-Cy7-anti-human CD3(BD Biosciences, USA), PE-anti-human IFN-γ (BD Biosciences, USA), BV421-anti-human CD45RA (BD Biosciences, USA), APC-anti-human-CD62L (Biolegend, USA), Fixable Viability Stain 510 (BD Biosciences, USA). The stained cells were washed with Flow Cytometry Staining Buffer (eBioscience, USA) and fixed with IC Fixation Buffer (eBioscience, USA). Flow cytometry analyses were performed with a FACS Calibur flow cytometer (BD Biosciences, USA) and analyzed by FlowJo software.

### Histological staining

For immunohistological or immunofluorescence staining, paired EAT and SAT samples were fixed in 4% paraformaldehyde at 25 °C for 24 hours and embedded in paraffin. Slides were sectioned in 5μm and blocked with 1% BSA PBS buffer and then stained with target antibodies and DAPI following routine procedures. The slides were scanned with a digital scanner (3D-HISTECH, Hungary). CaseViewer software was used for observation and statistics. For immunohistological statistics, 3 areas under the 20x field of view from each slide were randomly selected, and the average number of positive cells per mm^2^ was calculated (3 slides included for each sample). Used antibodies were as follows: human CD3 antibody (Servicebio, China), human CCL5 antibody (R&D systems, USA), and human GZMK antibody (R&D systems, USA).

### Statistical analysis

Data processing and analyses were performed using SPSS 22.0, GraphPad Prism, and R. Normality were evaluated by the Shapiro-Wilk test. Differences were evaluated using Student’s t-test and *P* < 0.05 was considered statistically significant unless indicated otherwise.

## Results

### Integrated bioinformatics analyses revealed pro-inflammatory characteristics of EAT

**Figure 2:**
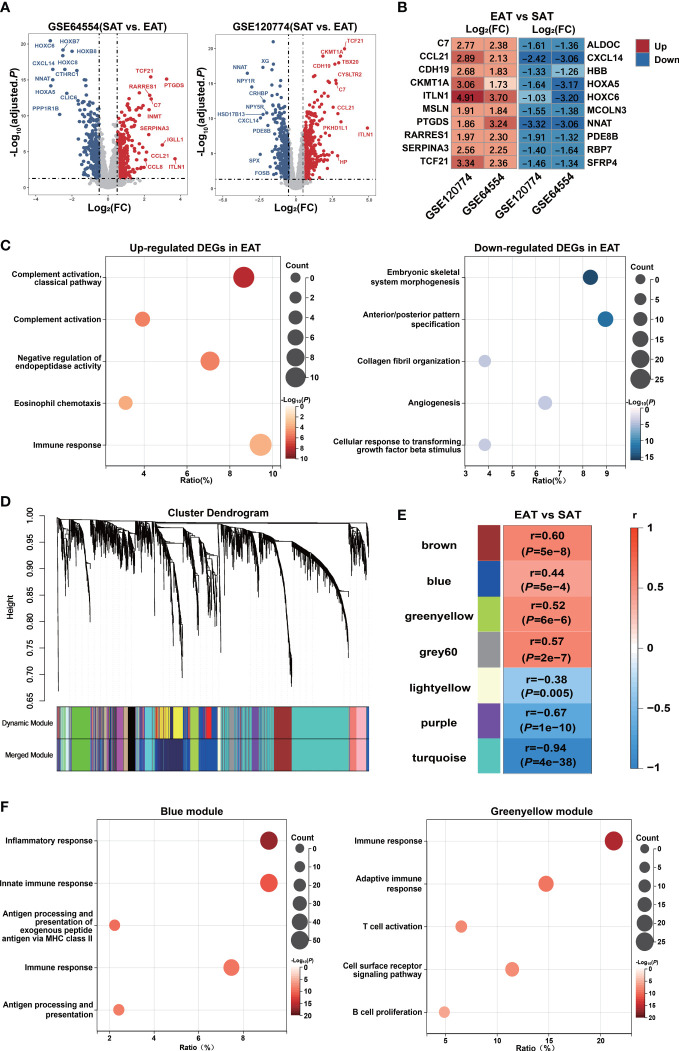
Bioinformatics analyses reveal pro-inflammatory characteristics and key genes of EAT. **(A)** Volcano plot of DEGs between EAT and SAT in GSE64554 and GSE120774. **(B)** Top 10 up- and down-regulated DEGs identified by RRA method. **(C)** GO-BP functional enrichment analyses of up- and down-regulated DEGs in EAT compared to SAT. **(D)** Cluster dendrogram of WGCNA. **(E)** WGCNA key modules and EAT/SAT phenotype correlation. **(F)** GO-BP functional enrichment analyses of WGCNA-identified blue and greenyellow gene modules.

The DEGs between EAT and SAT in GSE64554 and GSE120774 were identified separately and shown in **Figure 2A**. Next, we applied the RRA algorithm to integrate DEGs of the two datasets and obtain a more comprehensive DEGs list. The RRA method identified 131 genes that were up-regulated in EAT compared to SAT, while 159 genes were down-regulated. DEGs identified by RRA presented significant differences (adjusted *P* value<0.05 and |log_2_FC| ≥0.5) in at least one dataset, most of which (90%) showed consistent expressing trends across datasets. The top10 up- and down-regulated DEGs recognized by RRA were shown in **Figure 2B**. Next, we applied Gene Ontology (GO) enrichment analysis on the DEGs identified by RAA that were up- and down-regulated in EAT versus SAT to explore their potential functions, respectively. As shown in **Figure 2C**, the up-regulated DEGs in EAT were mainly enriched in complement activation and immune response, while the down-regulated DEGs were mainly related to embryonic skeletal system morphogenesis, suggesting immune activation in EAT compared to paired SAT. The PPI network of DEGs identified by STRING was further analyzed by cytoHubba to identify hub genes. As shown in **Figure S2** and **Table S5**, we obtained the top 10 hub genes including *COL1A1*, *FGF2*, *BGN*, *C3*, *TIMP1*, *CD44*, *POSTN*, *COL3A1*, *CCL2* and *APOB*.

Further, we applied the WGCNA method to identify immune-related key gene modules associated with EAT. By filtering the expression profiles of the top 25% variance in all EAT and SAT samples, a total of 3869 highly variable genes were included in WGCNA. Then, filtered genes were clustered into 18 different modules based on WGCNA clustering (**Figure 2D**). The correlation analyses between all modules and EAT/SAT phenotype were carried out and 7 modules were found to be significantly associated with the EAT/SAT phenotype (**Figure 2E**). Functional enrichment analyses suggested that the blue and greenyellow modules were closely related to immune response (**Figure 2F**). The overlap of DEGs and the two modules were identified and 9 key genes were obtained for further analyses. Of the 9 key genes, all were up-regulated DEGs in EAT and listed in **Table S6**, including *SLCO2B1*, *F13A1*, *C1QA*, *C1QB*, and *C1QC* from the blue module and *IGLL1*, *GZMK*, *CCL5*, and *SLC38A1* from the greenyellow module.

### Immune cell infiltration analyses showed a potential enrichment of lymphocytes in EAT

**Figure 3:**
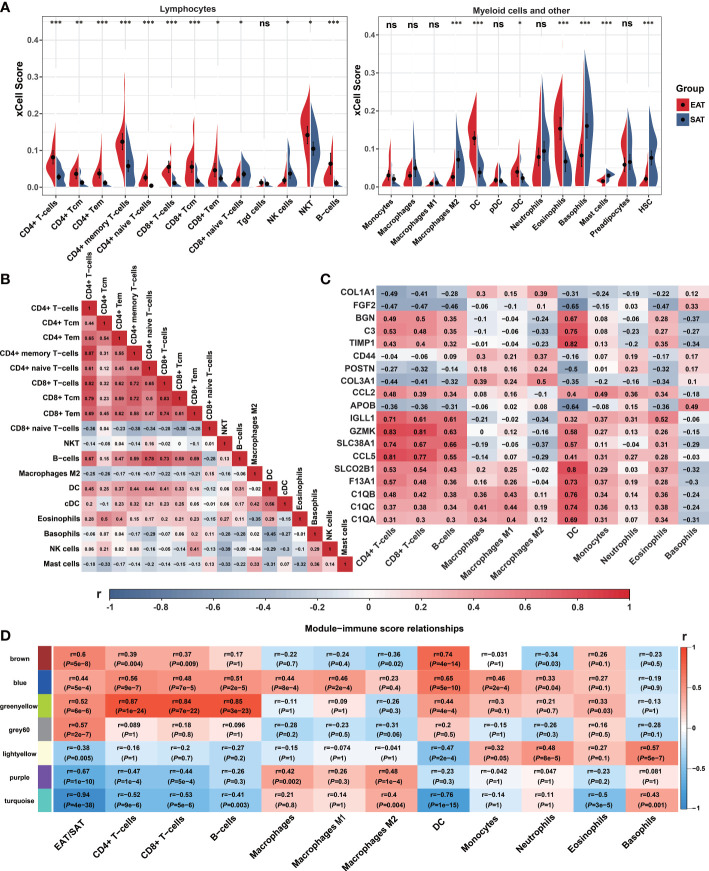
Immune cell infiltration and correlation analyses. **(A)** Violin charts of xCell immune infiltration score between EAT and SAT. **(B)** Correlation matrix of immune cell subtypes (Pearson correlation coefficients are displayed in the box). **(C)** Correlation matrix of immune cell infiltration scores with 19 identified key genes (Pearson correlation coefficients are displayed in the box). **(D)** Correlation matrix of immune cell infiltration scores with WGCNA key modules. *P < 0.05, **P < 0.01, ***P < 0.001 and ns refers to no significance.

We used xCell to explore the differences in the immune cell landscape between EAT and SAT. As shown in **Figure 3A**, EAT was infiltrated by more lymphocytes and dendritic cells (DC), while the abundance of macrophages and M1 macrophages showed no significant difference. In SAT, M2 macrophages, basophils, and mast cells showed higher degrees of infiltration. The correlation between different cell subtypes was calculated to infer their potential interaction. In **Figure 3B**, CD4^+^ T cells and CD8^+^ T cells presented a strong positive correlation (r=0.82), indicating that the two subtypes of T cells had a consistent tendency of infiltration.

Next, we analyzed the correlation between the infiltrated immune cells with hub genes, key genes and key modules identified in EAT from the previous PPI network and WGCNA analyses. As shown in **Figure 3C**, the expression of *GZMK*, *CCL5*, *IGLL1*, and *SLC38A1* presented a strong positive correlation with CD4^+^ T cells, CD8^+^ T cells, and B cells, while the expression of *SLCO2B1*, *BGN*, *C3*, *TIMP1*, *C1QA*, *C1QB* and *C1QC* showed a strong positive correlation with DC. As shown in **Figure 3D**, the 7 key modules related to EAT and SAT obtained by WGCNA were all related to different subtypes of immune cells. In particular, the blue and greenyellow modules closely related to the immune process presented a strong positive correlation with lymphocyte abundance. The correlation coefficients between the greenyellow module and T or B cells were more than 0.8. Based on the above analyses, we concluded that EAT acts as a pro-inflammatory adipose tissue characterized by abundant lymphocyte infiltration compared with SAT.

### More activated T lymphocytes in EAT from HF patients

**Figure 4:**
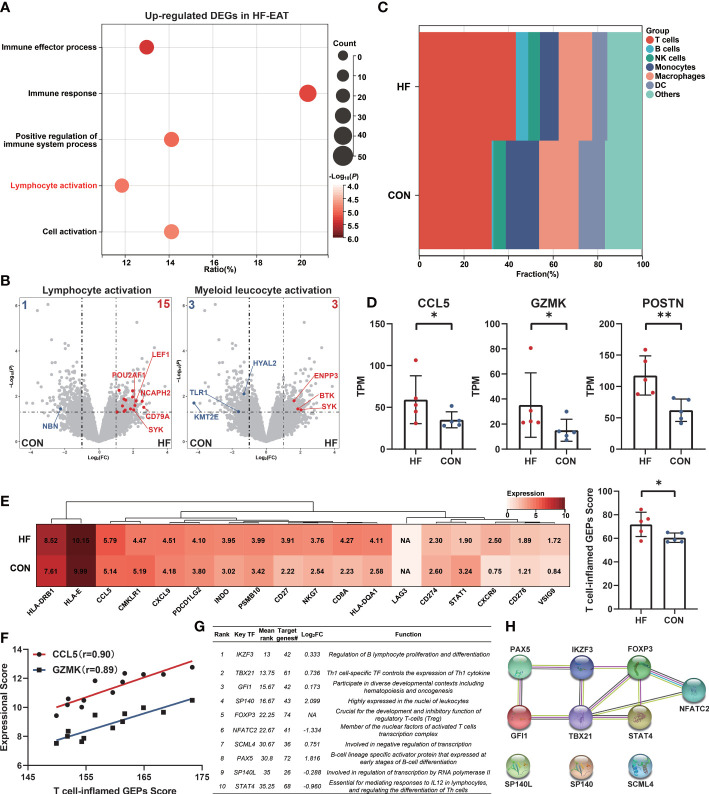
Amplified lymphocyte activation features in EAT of HF patients. **(A)** GO-BP functional enrichment analyses of up-regulated DEGs in EAT of HF patients. **(B)** “Lymphocyte activation” and “myeloid leucocyte activation” GO term genes in DEGs of HF-EAT. **(C)** Immune cell infiltration analyses of HF-EAT and control EAT by CIBERSORT. **(D)** Differentially expressed key genes in HF-EAT. **(E)** Expression of T cell-inflamed GEPs in HF-EAT and control EAT. **(F)** Correlation of *CCL5* and *GZMK* expression with T cell-inflamed GEPs score in GSE24425. **(G)** Top 10 potential key TFs of DEGs in HF-EAT identified by ChEA3 database. **(H)** PPI network of top 10 potential TFs. *P < 0.05, **P < 0.01.

To further explore the characteristics of EAT from HF patients, we analyzed a public dataset GSE192886 containing transcriptome profiles of EAT from 5 HF patients and 5 patients without HF as controls (CON). We obtained 196 up-regulated and 261 down-regulated DEGs in EAT from HF patients versus that from controls. Function enrichment analysis of up-regulated DEGs suggested immune activation, particularly lymphocyte activation in EAT from HF patients (**Figure 4A**). Up-regulated DEGs were mainly enriched in the lymphocyte activation pathway relative to the myeloid leukocyte activation pathway (**Figure 4B**). Next, we used CIBERSORT to compare the immune cell composition between EAT from HF patients and non-HF controls. As shown in **Figure 4C**, the frequencies of T cells and B cells were higher in EAT from HF patients compared to non-HF controls, indicating lymphocyte activation as the hallmark of EAT from HF patients.

We examined the expression levels of genes associated with the inflammatory characteristics of EAT (10 hub genes and 9 key genes identified above). As shown in **Figure 4D** and **Figures S3**, **S4** (Mann-Whitney test), of these genes (*IGLL1* not included), the expression of *CCL5*, *GZMK*, and *POSTN* showed a further increase in EAT from HF patients indicating an enhanced degree of inflammation and fibrosis while *CCL5* and *GZMK* presented the strongest positive correlation with infiltrated T lymphocytes in previous analyses (**Figure 3C**). Next, we examined the expressions of T cell-inflamed gene expression profiles (GEPs) between EAT from HF patients and non-HF controls. T cell-inflamed GEPs (composite genes listed in **Table S7**) has been reported to be associated with inflammatory T-lymphocyte infiltration and prediction of sensitivity to immunotherapy in tumors (26, 27). As shown in **Figure 4E** (Mann-Whitney test), the expressions of T cell-inflamed GEPs were higher in EAT of HF patients, providing further evidence of an enhanced T-lymphocyte response. In addition, the expression of *CCL5* and *GZMK* were also strongly positively correlated with T cell-inflamed GEPs in EAT and SAT samples from validation dataset GSE24425 (**Figure 4F**).

Next, we identified the potential key TFs regulating the phenotypic transition of EAT from HF patients using the ChEA3 database (28) (**Figure 4G**). The PPI network of top 10 predicted key TFs suggested a crucial role of lymphocyte-specific TFs in EAT of HF patients, especially for those were differentially expressed including TBX21, PAX5, NFATC2, and STAT4 (**Figure 4H**).

### Characteristics of TCR repertoires in EAT from HF patients

**Figure 5:**
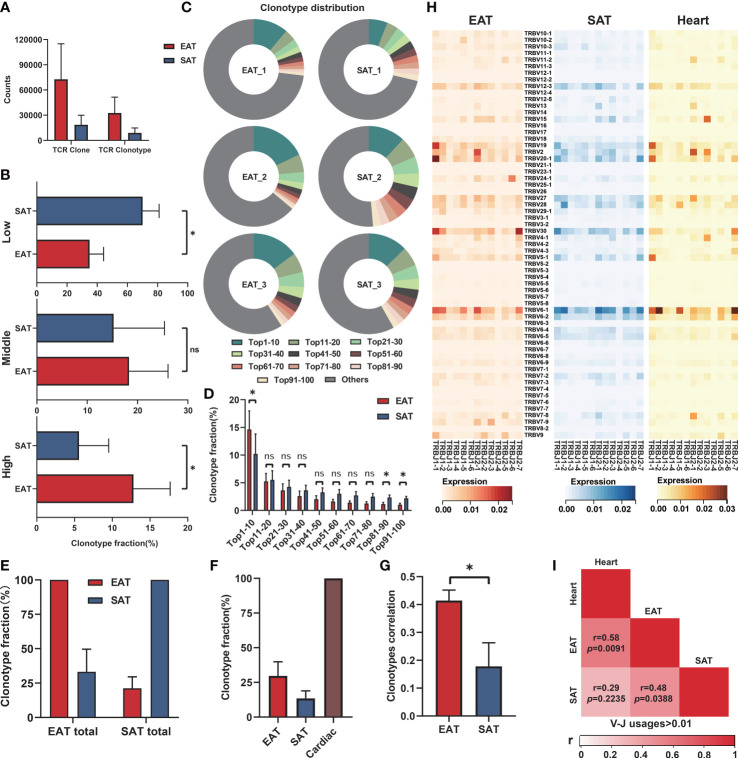
Characteristics of TCR repertoires in EAT. **(A)** TCR clone counts and TCR clonotype counts in EAT and paired SAT. **(B)** Fraction of low (proportion>0.1%), middle (proportion>0.5%) and high (proportion>1%) frequency TCR clonotypes between EAT and paired SAT. **(C, D)** The ratio and difference of the top 100 TCR clonotypes in EAT and paired SAT. **(E)** Total TCR clonotypes sharing between paired EAT and SAT. **(F)** Total cardiac TCR clonotypes sharing in paired EAT and SAT. **(G)** Spearman’s correlation of cardiac TCR clonotypes with paired EAT and SAT. **(H)** Heat map of V-J usage between EAT, paired SAT and heart. **(I)** Spearman’s correlation of V-J combination (average usage>0.01) in EAT, SAT and heart. *P < 0.05 and ns refers to no significance.

The numbers of TCR clones and TCR clonotypes were higher in EAT than in paired SAT from HF patients, indicating enhanced T lymphocyte infiltration in EAT (**Figure 5A**). Next, we compared the distribution of the low (fraction>0.1%), middle (fraction>0.5%), and high (fraction>1%) frequency TCR clonotypes between EAT and paired SAT. The results suggested the enrichment of highly expanded TCR clonotypes in EAT compared to paired SAT (**Figure 5B**). Accordingly, the proportion of top 10 TCR clonotypes was higher in EAT than in SAT (**Figures 5C, D**). These results suggested that T lymphocytes from EAT of HF patients exhibited higher clonal expansion than those from SAT. TCR clones with high frequency in EAT were listed and evaluated by antigen matching analysis *via* the IEDB database (**Tables S8**, **S9**).

A relatively low proportion of shared TCR clonotypes was observed between EAT and paired SAT (**Figure 5E**). However, the degree of TCR clonotype sharing between cardiac tissue and EAT was higher than that between cardiac tissue and SAT (**Figure 5F**). Further, we found the Spearman’s correlation coefficients between frequencies of TCR clonotypes in cardiac tissue and paired EAT was higher compared to that of SAT (**Figure 5G**). Next, we examined the usages of TRBV-TRBJ fragments in EAT, SAT, and cardiac tissue (**Figure 5H**). For the frequency distribution of V-J fragments with an average frequency >1% in the heart (19 V-J fragments ranked in **Figure S5**), the correlation between cardiac tissue and EAT was greater than that between EAT and SAT while no obvious correlation was observed between cardiac tissue and SAT (**Figure 5I**). Thus, the above results suggested a similar antigenic microenvironment between the heart and adjacent EAT.

### Characteristics of T lymphocytes functional phenotypes in EAT from HF patients

**Figure 6:**
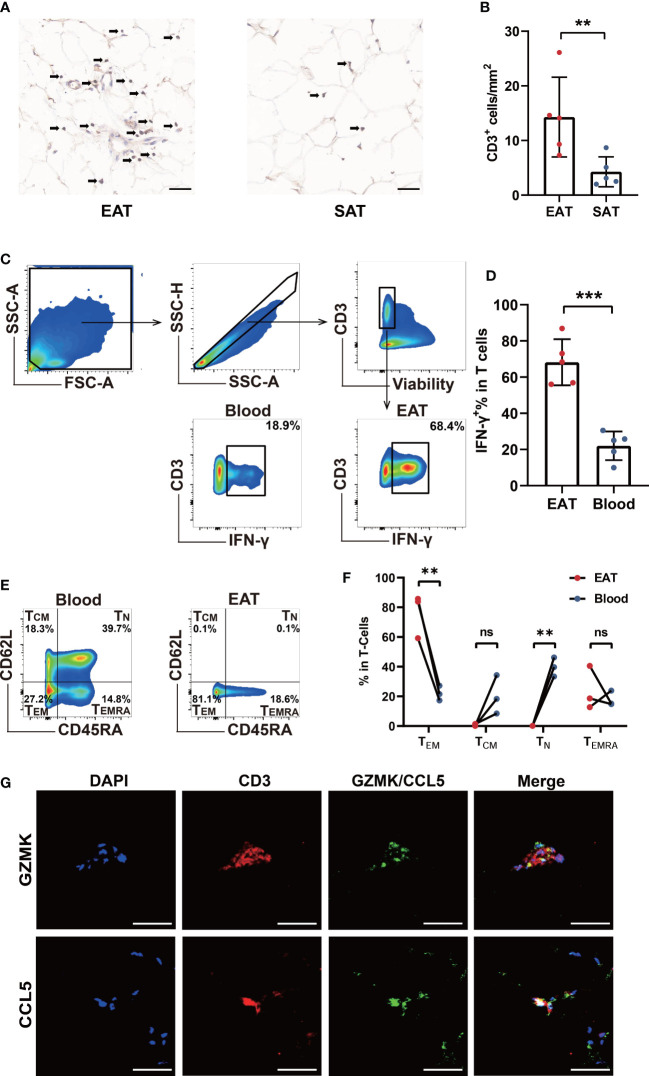
Verification of T cells infiltration and key molecules in EAT. **(A, B)** CD3-specific immunohistochemical staining in EAT and SAT. **(C, D)** Gating strategy and representative flow cytometry results of IFN-γ^+^ T lymphocytes in EAT. **(E, F)** Representative flow cytometry results for proportion of T lymphocytes memory subtypes in EAT. **(G)** Representative fluorescent staining images of CCL5 and GZMK with CD3 from EAT of HF patients (scale: 50 μm). **P < 0.01, ***P < 0.001 and ns refers to no significance.

In order to verify the accumulation of T lymphocytes in EAT and the role of key genes, we collected EAT together with paired SAT and peripheral blood samples from HF patients undergoing heart transplantation. Immunohistomical staining showed abundant CD3-positive T lymphocytes in EAT compared to SAT (**Figures 6A, B**). Further flow cytometry showed that enriched T lymphocytes in EAT were mainly composed of T_EM_ expressing high levels of IFN-γ (**Figures 6C–F**). Further immunofluorescence staining results confirmed that CCL5 and GZMK were co-localized with CD3-positive T lymphocytes (**Figure 6G**). Taken together, we concluded that EAT from HF patients were populated by inflammatory T_EM_ cells expressing high levels of effector molecules including IFN-γ, CCL5, and GZMK and thus contributing to EAT pro-inflammatory conversion in HF patients.

## Discussion

Previous understanding of the pro-inflammatory characteristics of EAT was limited to the paracrine and endocrine effects of adipokines and cytokines produced by EAT. The profiles of immune cells in EAT have rarely been elucidated. A pioneering work by Hirata et al. (10) suggested that macrophages in EAT from patients with coronary artery disease tend to be polarized towards the pro-inflammatory M1 phenotype. Recently, Vyas et al. (9) found that EAT was highly enriched in adaptive immune cells. Given the relatively simple cellular composition of adipose tissue, integrated bioinformatics analyses based on the high-throughput array or sequencing data could expand our knowledge of the roles and characteristics of immune cells in EAT.

Based on our analyses, EAT was enriched in immune activation-related pathways and T lymphocytes compared to paired SAT and this trait was more pronounced in EAT from HF patients. Further, we used high-throughput TCR sequencing to explore the characteristics of TCR repertoires in EAT and found enrichment of highly expanded TCR clonotypes in EAT from HF patients. In addition, we found a higher degree of TCR clonotypes sharing between EAT and paired cardiac tissue from HF patients relative to SAT, suggesting a similar antigenic microenvironment between the heart and adjacent EAT. Furthermore, we demonstrated the dominance of pro-inflammatory IFN-γ^+^ effector memory T lymphocytes in EAT from HF patients. Considering our previous work has revealed a tissue-specific T-cell response predominated by clonally expanded Th1 and cytotoxic CD8^+^T lymphocytes in failing human hearts (29), the present work may provide further evidence of a similar immune microenvironment at the cellular level between EAT and heart.

CCL5 and GZMK may be the key effector molecules of T lymphocytes in EAT. GZMK produced by cytotoxic T lymphocytes mediates cell death by displaying tryptase-like activity (30). It has been reported that GZMK assists transcellular diapedesis of T_EM_ by inducing the expression of *ICAM1* in endothelial cells (31). CCL5 belongs to the C-C motif chemokine family and binds to its receptor CCR5 (32). CCL5 can be produced by a variety of cells including T lymphocytes, macrophages, fibroblasts, and epithelial cells, and regulates the migration of T lymphocytes and monocytes (32). CCL5 expression was found to be higher in visceral adipose tissue (VAT) compared to SAT and positively correlated with CD3 and CD11b expression (33), while Zhou et al. (34) further identified CD8^+^ T lymphocytes as the major cellular sources of CCL5 in the VAT of obese mice. A recent study showed that clonally expanded GZMK^+^CD8^+^ T cells producing a high level of CCL5 may promote the recruitment of pro-inflammatory immune cells and elevate tissue inflammation (35). Taken together, GZMK and CCL5 may act as key effectors in mediating the adaptive immune response of T lymphocytes in EAT of HF patients.

Existing evidence suggest that increased EAT volume was associated with an increased risk of HF with preserved ejection fraction (HFpEF) (36). However, EAT volume was reduced in HF patients with reduced ejection fraction (HFrEF) (37). In HFpEF patients, increased EAT volume was associated with higher concentrations of troponin T, hs-CRP, IL-6, and increased risk of cardiovascular death and hospitalization, while these associations were reversed in HFrEF patients (6). The reason for the discrepancy may be due to the increased intra-myocardial fat energy requirement in patients with HFrEF because of the progression to cachexia state (38). The reduction of EAT may exacerbate the progression of HFrEF by diminishing the ability of the myocardium to nourish from adjacent EAT. Since the pro-inflammatory conversion of EAT often precedes the clinical diagnosis of HF (4), the specific causal relationship between EAT and different types or stages of HF remains unclear.

To conclude, EAT of HF patients was characterized by pronounced immune activation, particularly by the accumulation of IFN-γ^+^ T_EM_ and a generally higher degree of TCR clonotypes sharing with paired cardiac tissue. GZMK and CCL5 may act as the key effector molecules of T lymphocytes in EAT of HF patients. Our study has certain limitations. First, we used expression profiles from public datasets to infer immune cell infiltration scenarios of EAT, which may have discordance with actual situations. Second, the samples were obtained from end-stage HF patients and the sample size was small in the validation experiments. More detailed exploration of immune cell profiles in EAT from different stages of HF patients is deserved in the future.

## Conclusion

EAT of HF patients was characterized by pronounced immune activation of clonally expanded IFN-γ^+^ T_EM_ and a generally higher degree of TCR clonotypes sharing with paired cardiac tissue.

## Data availability statement

The datasets presented in this study can be found in online repositories. The names of the repository/repositories and accession number(s) can be found below: PRJNA925305 (SRA).

## Ethics statement

The studies involving human participants were reviewed and approved by Medical Ethics Committee of Wuhan Union Hospital of Huazhong University of Science and Technology. The patients/participants provided their written informed consent to participate in this study.

## Author contributions

XC, X-ZZ, X-LC, and T-TT contributed to experiments design, data analyses, and manuscript writing. SZ and Q-LL contributed to reviewing the bioinformatics analyses. NX, S-FN, MZ, and Z-FZ contributed to reviewing the manuscript. Z-HZ contributed to reviewing and revising the manuscript. N-GD contributed to collecting clinical samples. All authors contributed to the article and approved the submitted version.
