# Supplementary material for: T lymphocyte characteristics and immune repertoires in the epicardial adipose tissue of heart failure patients
Source: Front Immunol. 2023 Mar 7;14:1126997. doi: 10.3389/fimmu.2023.1126997 (PMC10027920; doi:10.3389/fimmu.2023.1126997)
Supplement: Supplementary file 1 [file DataSheet_1.pdf]

T Lymphocyte Characteristics and Immune Repertoires in the Epicardial Adipose Tissue of Heart Failure Patients

Supplementary Material

Xu-Zhe Zhang[1,2,3†], Xian-Li Chen[1,2,3†], Ting-Ting Tang[1,2,3†], Si Zhang[1,2,3], Qin-Lin Li[1,2,3], Ni Xia[1,2,3], Shao-Fang Nie[1,2,3], Min Zhang[1,2,3], Zheng-Feng Zhu[1,2,3], Zi-Hua Zhou[1,2,3*], Nian-Guo Dong[4*] and Xiang Cheng[1,2,3*]

[1]Department of Cardiology, Union Hospital, Tongji Medical College, Huazhong University of Science and Technology, Wuhan 430022, China.

[2]Hubei Key Laboratory of Biological Targeted Therapy, Union Hospital, Tongji Medical College, Huazhong University of Science and Technology, Wuhan 430022, China.

[3]Hubei Engineering Research Center for Immunological Diagnosis and Therapy of Cardiovascular Diseases, Union Hospital, Tongji Medical College, Huazhong University of Science and Technology.

[4]Department of Cardiovascular Surgery, Union Hospital, Tongji Medical College, Huazhong University of Science and Technology, Wuhan 430022, China

†These authors contributed equally to this work.

***Correspondence:**
Xiang Cheng, nathancx@hust.edu.cn; Nian-Guo Dong, dongnianguo@hotmail.com; Zi-Hua Zhou, zhouzihua@hust.edu.cn.

1 Supplementary Figures

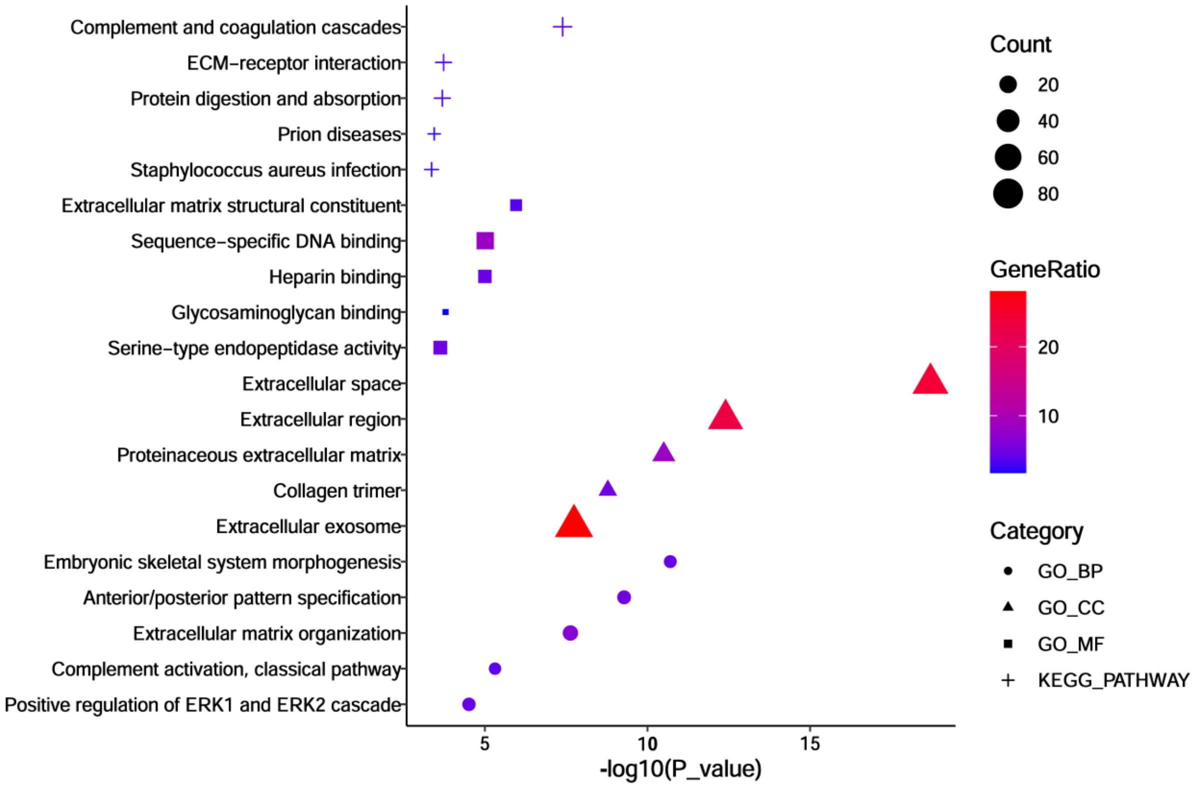

Supplementary Figure 1. Functional annotations of all DEGs (EAT vs. SAT) recognized by RRA method.

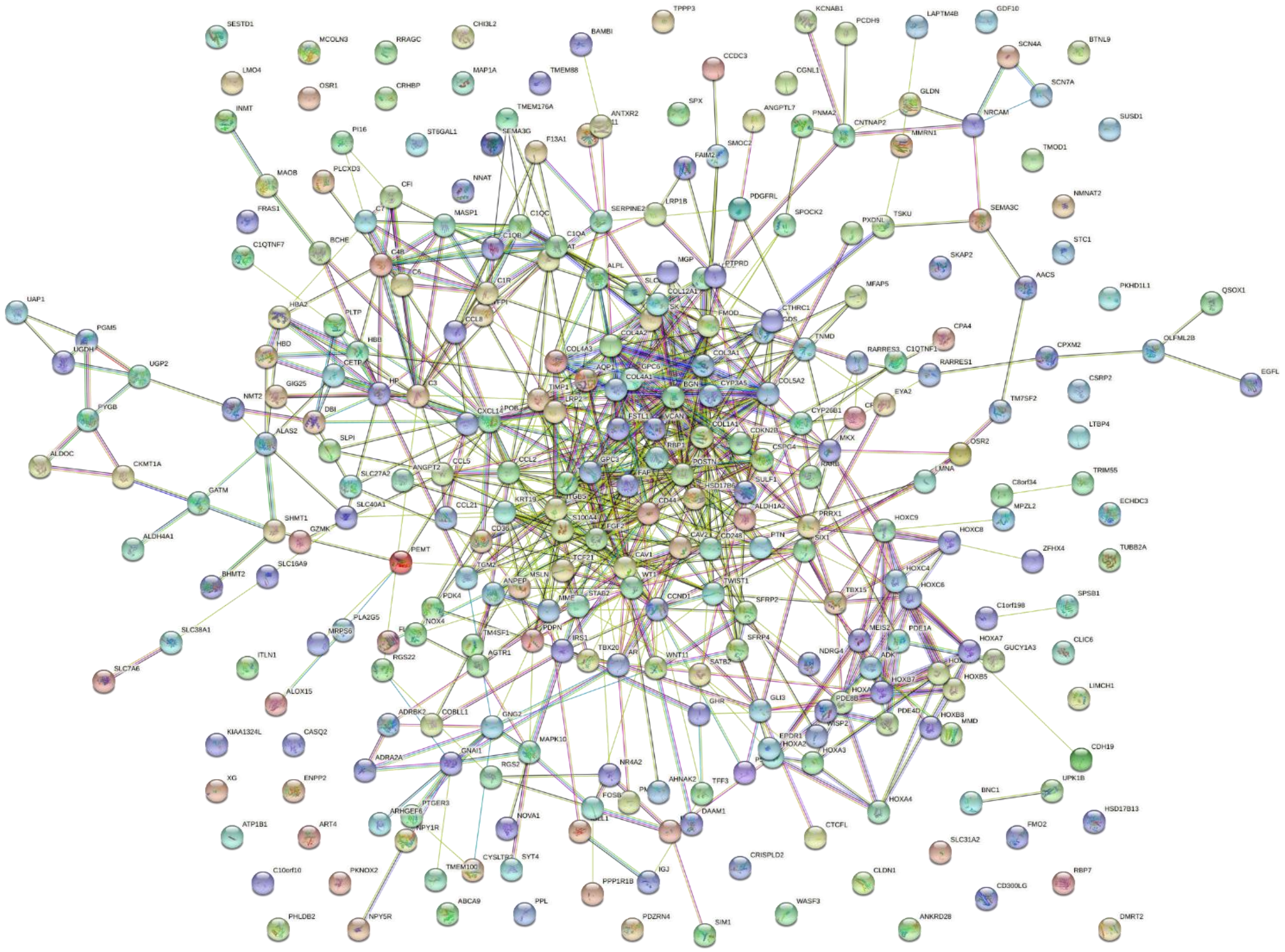

Supplementary Figure 2 PPI network of all DEGs (EAT vs. SAT) recognized by RRA method.

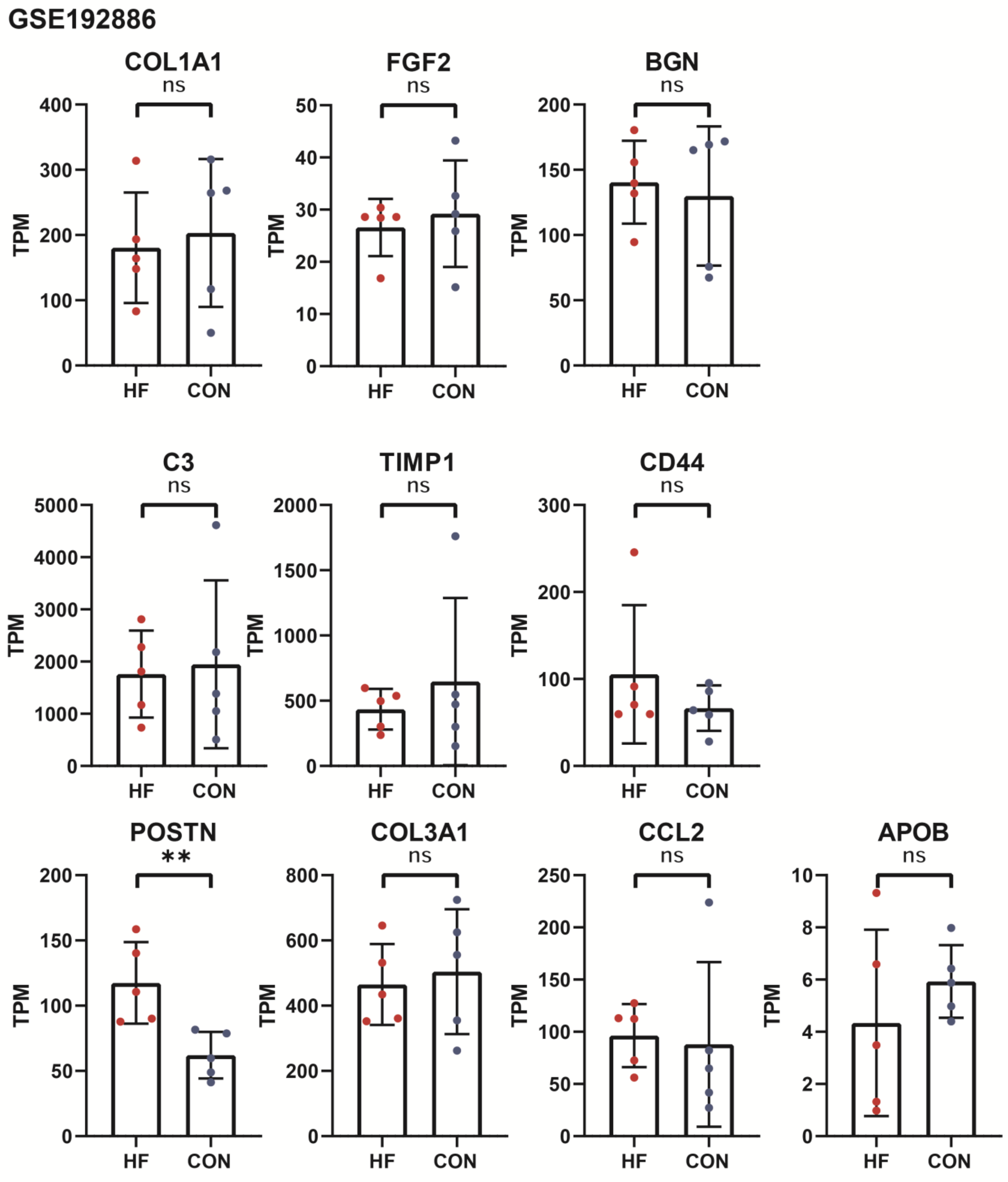

Supplementary Figure 3 Expressional differences of PPI network-identified hub genes between HF-EAT and control EAT in dataset GSE192886.

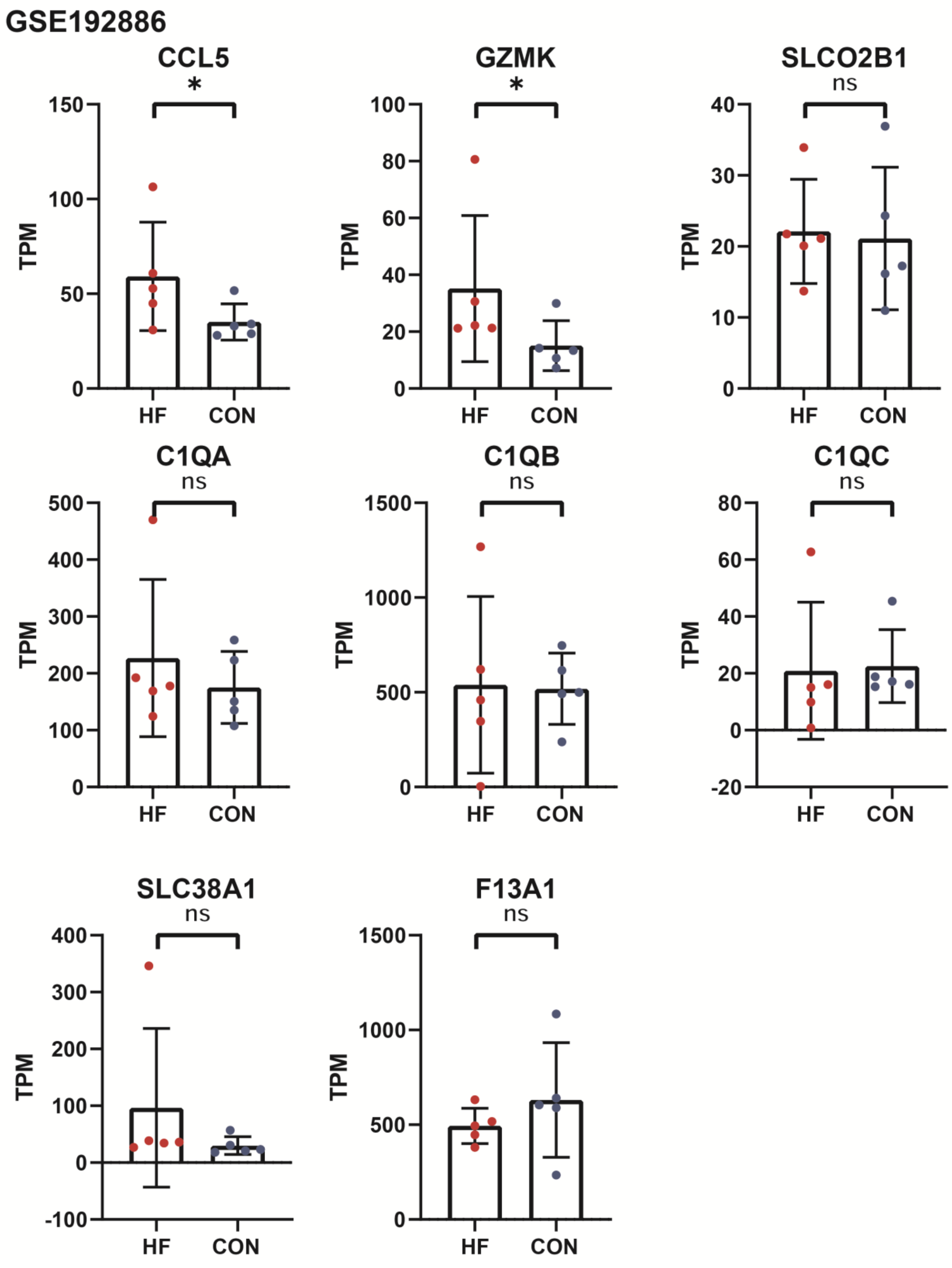

Supplementary Figure 4 Expressional differences of WGCNA-identified key genes between HF-EAT and control EAT in dataset GSE192886.

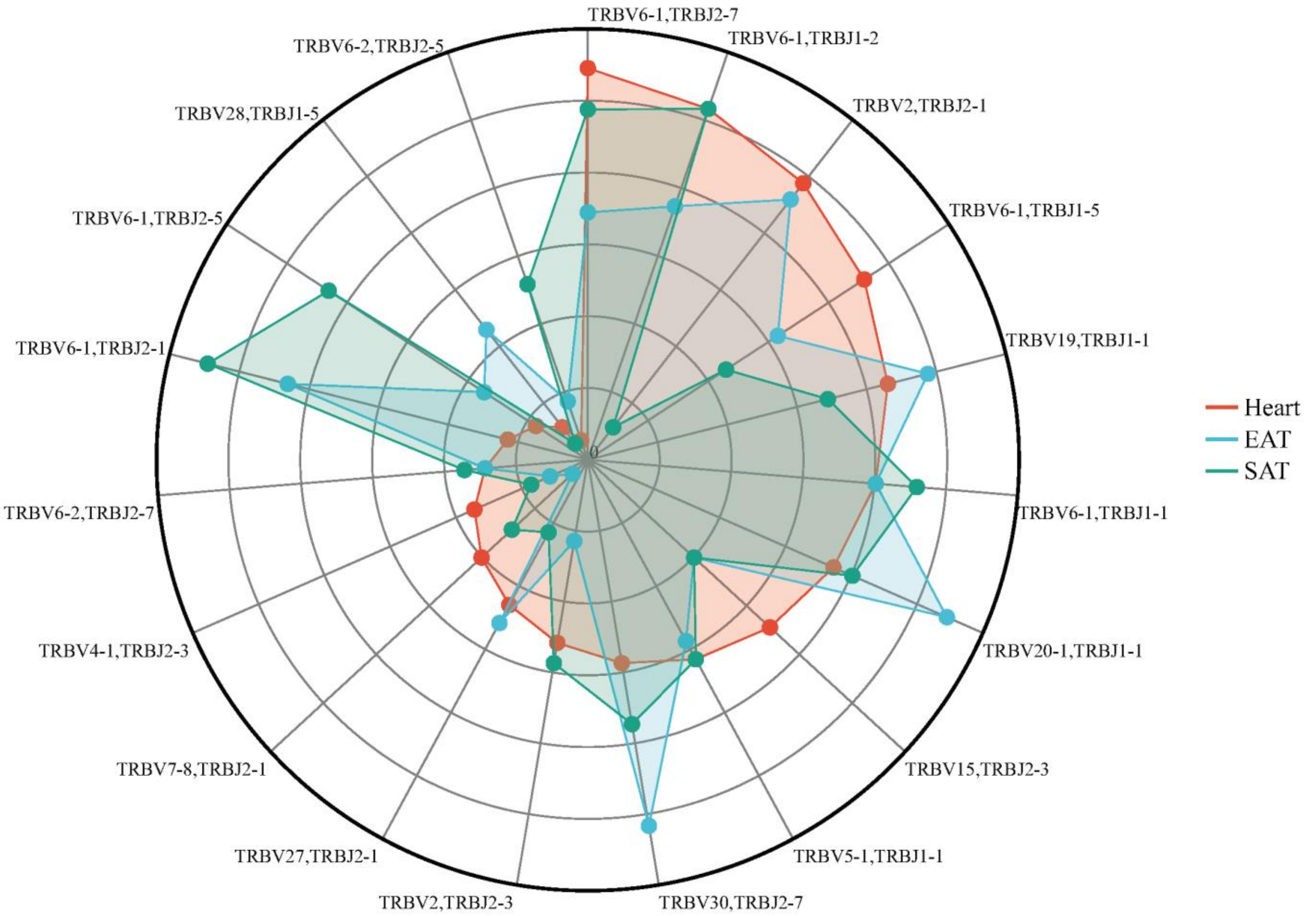

Supplementary Figure 5 The overlap radar chart of the TRBV-TRBJ fragments usages rank in EAT, SAT, and the heart (V-J fragments with an average frequency >1% in the heart).

2 Supplementary Tables

Table S1 Clinical characteristics of involved sample origin in GSE64554.

GSE64554	EAT	SAT
No.	23	23
Disease(CAD/Control),no.	13/10	13/10
Age, surgery (y), median (IQR)	69(52-73)	

Table S2 Clinical characteristics of involved sample origin in GSE120774.

GSE120774	EAT	SAT
No.	19	17
Disease(CAD/Control),no.	9/10	9/8
Sex (male/female), no.	11/8	10/7

Table S3 Clinical characteristics of involved sample origin in GSE192886.

GSE192886	HF	Non-HF
No.	5	5
Sex (male/female), no.	2/3	3/2
Age(y)	67.8±5.0	60.8±8.1
BMI, kg/m^2	23.8±4.6	25.0±5.5
LVEF(%)	44.2±10.2	62.0±4.8

Table S4 Overview of clinical subjects involved in validation experiments.

Subject	Etiology	Sample	Analysis	Age	Sex	LVEDD(cm)	LVEF (%)	NYHA
P1	ICM	E/S/H	TCR-seq/IHC	36	M	6.8	28	IV
P2	ICM	E/S/H	TCR-seq	56	M	6.6	21	IV
P3	ICM	E/S/H	TCR-seq	72	M	7.3	27	IV
P4	DCM	E/S	IHC	52	M	7.3	22	IV
P5	DCM	E/S	IHC	36	M	9.6	10	IV
P6	DCM	E/S/B	IHC/FC	59	M	10.3	25	IV
P7	DCM	E/S/B	IHC/FC	58	M	9.4	18	IV
P8	DCM	E/B	FC	44	F	6.7	21	IV
P9	DCM	E/B	FC	44	F	5.6	20	IV
P10	ICM	E/B	FC	66	M	6.8	21	IV
P11	DCM	E/B	FC	52	M	7.6	22	IV
P12	ICM	E/B	FC	56	M	6.7	25	IV
P13	DCM	E/B	FC	45	M	8.1	19	IV

Abbreviations: B, blood; DCM, dilated cardiomyopathy; E, EAT; F, female; FC, flow cytometry; H, heart; ICM, ischemic cardiomyopathy; IHC, immunohistochemistry; LVEDD, left ventricular end-diastolic dimension; LVEF, left ventricular ejection fraction; M, male; NYHA, NYHA classification of cardiac function; S, SAT.

Table S5 Top-10 hub genes of DEGs recognized by CytoHubba (ranked by degree).

Gene Symbol	$Log_2FC^†$	RRA score	Description
COL1A1	-1.644/-0.619	<0.05	Collagen Type I Alpha 1 Chain
FGF2	-0.698/-0.541	<0.05	Fibroblast Growth Factor 2
BGN	1.002/0.843	<0.001	Biglycan
C3	0.875/1.063	<0.01	Complement C3
TIMP1	1.003/1.278	<0.01	TIMP Metallopeptidase Inhibitor 1
CD44	-0.630/-0.676	<0.05	CD44 Antigen
POSTN	-0.228/-1.444	<0.05	Periostin
COL3A1	-0.976/-0.655	<0.05	Collagen Type III Alpha 1 Chain
CCL2	1.984/0.192	<0.05	C-C Motif Chemokine Ligand 2
APOB	-0.607/-1.435	<0.05	Apolipoprotein B

† Log_2FC from GSE64554/GSE120774, "+" for up-regulated in EAT, "-" for up-regulated in SAT.

Table S6 Nine key genes recognized by WGCNA.

Gene Symbol	Log_2FC[†]	RRA score	Description
IGLL1	3.000/0.102	<0.01	Immunoglobulin Lambda Like Polypeptide 1
GZMK	1.097/0.857	<0.05	Granzyme K
SLC38A1	0.687/0.963	<0.05	Solute Carrier Family 38 Member 1
CCL5	1.471/0.347	<0.05	C-C Motif Chemokine Ligand 5
SLCO2B1	1.570/1.060	<0.01	Solute Carrier Organic Anion Transporter Family Member 2B1
F13A1	1.065/0.665	<0.05	Coagulation Factor XIII A Chain
C1QA	0.620/0.596	<0.05	Complement C1q A Chain
C1QB	1.043/0.639	<0.05	Complement C1q B Chain
C1QC	0.901/0.614	<0.05	Complement C1q C Chain

† Log_2FC from GSE64554/GSE120774, “+” for up-regulated in EAT, “-” for up-regulated in SAT.

Table S7 T cell-inflamed gene expression profiles (GEPs) and their function.

Gene Symbol	Description	Function
CCL5	C-C Motif Chemokine Ligand 5	Chemokines for initiation of inflammation
CD27	CD27 Molecule	T-Cell Activation
CD274	PD-L1	Immune inhibitory receptor ligand
CD8A	CD8a Molecule	T cell markers
CMKLR1	Chemerin Chemokine-Like Receptor 1	Chemokines for initiation of inflammation
CXCL9	C-X-C Motif Chemokine Ligand 9	Chemokines for initiation of inflammation
CXCR6	C-X-C Motif Chemokine Receptor 6	Chemokines for initiation of inflammation
HLA-DQA1	Major Histocompatibility Complex, Class II, DQ Alpha 1	Antigen presentation
HLA-DRB1	Major Histocompatibility Complex, Class II, DR Beta 1	Antigen presentation
HLA-E	Major Histocompatibility Complex, Class I, E	Antigen presentation
INDO	Indoleamine 2,3-Dioxygenase 1	Immunomodulatory factors
LAG3	Lymphocyte Activating 3	Immunomodulatory factors
NKG7	Natural Killer Cell Granule Protein 7	NK cell activity
PDCD1LG2	Programmed Cell Death 1 Ligand 2	Immune inhibitory receptor ligand
PSMB10	Proteasome 20S Subunit Beta 10	Proteolytic activity
STAT1	Signal Transducer And Activator Of Transcription 1	IFN-gamma activation
VSTM3	T Cell Immunoreceptor With Ig And ITIM Domains	Immunomodulatory factors

Table S8 TCR clones with high frequency in EAT.

Patient	Fraction	CDR3 amino acid sequence	TRBV	TRBD	TRBJ
P1	0.0408	CASSGLADEQFF	TRBV2	TRBD2	TRBJ2-1
P1	0.0154	CASSVDPSTSSGETQYF	TRBV9	TRBD2	TRBJ2-5
P1	0.0136	CASSIAQLYGYTF	TRBV19	TRBD1	TRBJ1-2
P1	0.0102	CASSVQDTNTEAFF	TRBV2	TRBD1	TRBJ1-1
P2	0.0342	CSARRGDRGLWDTEAFF	TRBV20-1	TRBD1	TRBJ1-1
P2	0.0222	CASSSRRSDTGTDTQYF	TRBV7-9	TRBD1	TRBJ2-3
P2	0.0202	CASSATASTEAFF	TRBV9	TRBD1	TRBJ1-1
P2	0.0186	CAWSRGLAGFSTDTQYF	TRBV30	TRBD2	TRBJ2-3
P2	0.0178	CSAPNRDRGTKEQFF	TRBV20-1	TRBD1	TRBJ2-1
P2	0.0170	CASSVDTGELFF	TRBV9	TRBD1	TRBJ2-2
P2	0.0153	CASSDGDGGYNEQFF	TRBV7-9	TRBD2	TRBJ2-1
P2	0.0112	CATKSEREGNGELFF	TRBV15	TRBD1	TRBJ2-2
P2	0.0104	CATSRDWRNEQFF	TRBV15	TRBD2	TRBJ2-1
P2	0.0102	CSAIRDNSDGYTF	TRBV29-1	TRBD1	TRBJ1-2
P3	0.0387	CATSDFRSGANVLTF	TRBV24-1	TRBD2	TRBJ2-6
P3	0.0241	CATQTSGTANTEAFF	TRBV19	TRBD1	TRBJ1-1
P3	0.0138	CASRTGGLYQPQHF	TRBV10-2	TRBD1	TRBJ1-5
P3	0.0126	CASSLVLASRETQYF	TRBV7-8	TRBD2	TRBJ2-5
P3	0.0122	CSATADSATNEKLFF	TRBV20-1	TRBD1	TRBJ1-4
P3	0.0112	CAWSDESYRGGTEAFF	TRBV30	TRBD1	TRBJ1-1
P3	0.0107	CAWSNRAGAMNTEAFF	TRBV30	TRBD1	TRBJ1-1

Table S9 CDR3 amino acid sequence of TCR clones with high frequency in EAT matching in IEDB database (Matching score>0.96).

CDR3 amino acid sequence	Matching score	Matched sequence	Organism	Antigen
ASSATASTEAF	0.9752	ASSATTSTEAF	SARS-CoV2	orf1ab polyprotein
ASSVDTGELF	0.9714	ASSMDTGELF	Human herpesvirus 5 strain AD169	55kDa immediate-early protein 1
ASSVDTGELF	0.9675	ASSVDVGELF	SARS-CoV2	membrane glycoprotein
ASSVDTGELF	0.9660	ASSVNTGELF	SARS-CoV2	surface glycoprotein, orf1ab polyprotein
ASSGLADEQF	0.9644	ASSGLGDEQF	Influenza A virus,Human herpesvirus 5	nucleoprotein, HCMVUL83
ASSVDTGELF	0.9629	ASSVSTGELF	SARS-CoV2	orf1ab polyprotein
ASSVDTGELF	0.9625	ASSSDTGELF	SARS-CoV2	orf1ab polyprotein
ATSDFRSGANVLT	0.9620	ASSEFRSGANVLT	SARS-CoV2	Spike glycoprotein
ASSATASTEAF	0.9617	ASSSTANTEAF	SARS-CoV2	ORF7b
ASSATASTEAF	0.9606	ASSATGATEAF	SARS-CoV2	orf1ab polyprotein
